# Osteoporosis and fractures in systemic vasculitides: a systematic review and meta-analysis

**DOI:** 10.3389/fimmu.2025.1545546

**Published:** 2025-03-17

**Authors:** Angelo Fassio, Alvise Berti, Alessandro Mantovani, Giovanni Adami, Francesco Pollastri, Davide Gatti, Riccardo Bixio, Valeria Messina, Maurizio Rossini, Davide Bertelle, Eugenia Bertoldo, Isotta Galvagni, Roberto Bortolotti, Ombretta Viapiana

**Affiliations:** ^1^ Rheumatology Unit, University of Verona, Verona, Italy; ^2^ Center for Medical Sciences (CISMed), Department of Cellular, Computational, and Integrative Biology (CIBIO), University of Trento, Trento, Italy; ^3^ Unit of Rheumatology, Santa Chiara Regional Hospital, Azienda Provinciale per i Servizi Sanitari (APSS), Trento, Italy; ^4^ Section of Endocrinology, Diabetes and Metabolism, Department of Medicine, University and Azienda Ospedaliera Universitaria Integrata of Verona, Verona, Italy; ^5^ Rheumatology Section, Department of Medicine, Azienda Ospedaliera Friuli Occidentale, Pordenone, Italy

**Keywords:** vasculitis < rheumatic diseases, osteoporosis, fractures - bone, giant cell (temporal) arteritis, ANCA - associated vasculitis, glucocorticoids, DEXA - duel-energy X-ray absorptiometry, Takayasu ‘ s arteritis

## Abstract

**Background/aim:**

We performed a systematic review and meta-analysis of observational studies aimed at investigating the prevalence of osteoporosis and osteoporotic fractures in subjects affected by systemic vasculitides (SVs) as well as to explore their risk of osteoporosis and osteoporotic fractures when compared to healthy controls.

**Methods:**

Scopus, Web of Science and PubMed were systematically searched from inception to February 2024 for observational studies investigating the prevalence of osteoporosis and fragility fractures in adults with SVs. In addition, when available, we assessed the odd ratios (OR) of prevalent osteoporosis and fragility fractures amongst subjects with SVs vs. healthy controls. Data from eligible studies were extracted, and meta-analysis was performed using a random effects model to obtain ORs with 95% confidence intervals (CIs). Subgroup analyses and meta-regressions were also performed. This study was registered in Open Science Framework (DOI: https://doi.org/10.17605/OSF.IO/3G7RJ).

**Results:**

Forty studies with 23,358 individuals affected by SVs were included. The overall prevalence of osteoporosis and fragility fractures in the SV patients were respectively 14.64% (95%CI 12.21-18.89), and 17.08% (95%CI 11.42-24.78). The ORs for osteoporosis and fragility fractures in SV patients when compared with healthy controls were 2.92 (95%CI 1.72-4.98) and 2.39 (95%CI 1.34-4.26) respectively. The univariable meta-regression analysis showed a significant association between cumulative glucocorticoids’ dosage (total grams) and risk of prevalent osteoporosis (estimate = 0.0995, R^2^ = 0.24, p=0.0194).

**Conclusion:**

SVs are associated with an increased risk for osteoporosis and fragility fractures, suggesting that active vigilance and pre-emptive screening are recommended.

**Systematic review registration:**

https://archive.org/details/osf-registrations-3g7rj-v1.

## Background

Vasculitides represent a group of rare, systemic conditions histologically characterized by the infiltration of leukocytes and inflammation within the blood vessel walls. The Chapel-Hill Consensus Conference Nomenclature of Vasculitides (CHCC) organizes these diseases based on discernible features that differentiate various forms into distinct categories (1). The primary classification criterion is based on the predominant involvement of specific vessel types, namely large, medium, and small vessels.

The varying sizes of these vessel subsets reflect their functions and susceptibility to distinct variants of vasculitis (2). Large vessel vasculitides encompasses giant cell arteritis (GCA) and Takayasu Arteritis (TAK), while polyarteritis nodosa and Kawasaki Disease typically affect medium-sized blood vessels. Small-vessel vasculitides are primarily associated with antineutrophil cytoplasmic antibody (ANCA)-associated vasculitis (AAV), anti-glomerular basement membrane disease, cryoglobulinemic vasculitis, IgA vasculitis (Henoch-Schonlein), and hypocomplementemic urticarial vasculitis (anti-C1q vasculitis). Furthermore, this classification revision includes other forms of vasculitis, such as single-organ vasculitis or vasculitis involving vessels of any size (small, medium, and large) and type (arteries, veins, and capillaries).

The management of systemic vasculitis (SVs) is tailored to the specific vasculitis type but traditionally entails a regimen of high-dose glucocorticoids (GCs) either alone or alongside other immunosuppressive agents. Current guidelines support the use of high doses of GCs are generally used to induce remission in vasculitides, in the first months of therapy, while suggesting low-dose GCs therapy for maintenance of remission, during the follow-up.

Because of GCs, subjects with small and large vessel vasculitis face heightened fracture risks, in addition to other GC complications. However, GC-induced etiology is not the only risk factor of osteoporosis: chronic inflammation, nutritional inadequacy leading to vitamin D and calcium deficiency, impaired renal function, or other pharmacological interventions may increase the risk of impaired bone health (3).

Interestingly, over the past decade, there has been a notable increase in observational studies focusing on osteoporosis and fractures in idiopathic inflammatory vasculitides. Even if these studies globally showed an increase in osteoporosis prevalence in the different cohorts, different study designs, different populations (i.e. patients with large versus small vessels) and case ascertainment strategies may explain the heterogeneous findings among these different studies, often hampering a direct comparison among them and thus definitive conclusions.

Based on these considerations, we conducted a systematic review and meta-analysis to assess the prevalence of osteoporosis and osteoporotic fractures in individuals with SVs, as well as their risk for these complications compared to healthy controls.

## Materials and methods

### Search strategy and selection criteria

We systematically searched 3 large electronic databases (Scopus, Web of Science and PubMed) from database inception to 01 December 2024, using pre-defined key words, to identify observational studies examining the prevalence of osteoporosis amongst adult individuals with and without SVs, as well as the risk of prevalent osteoporosis amongst those with SVs when compared to healthy controls. The search strategy is reported in the **Supplementary Material**. We also reviewed references from original papers and review articles to identify further eligible studies not covered by our original database searches. This systematic review was performed according to the updated Preferred Reporting Items for Systematic Reviews and Meta-Analysis (PRISMA) statement (4). We also followed the reporting proposed by the Meta-analysis Of Observational Studies in Epidemiology (MOOSE) for the meta-analysis of these studies (5).

Studies were included in the meta-analysis if they met the following criteria: 1) original studies conducted in adults affected by systemic vasculitis; 2) all types of primary SVs as reported in the 2012 CHCC, including IgG4-RD.

Criteria for exclusion of the studies from the meta-analysis were as follows: 1) congress abstracts, case reports, reviews, practice guidelines; 2) studies which did not specifically report any data for the outcome of interest; 3) studies involving secondary forms and those with probable etiology. Searches was confined to randomized controlled trials, retrospective, longitudinal or cross-sectional studies. Studies with less than 10 subjects were excluded. Studies enrolling other forms of non-systemic vasculitides, such as isolated cutaneous or single-organ vasculitides were not included.

For all eligible studies we extracted data regarding the main characteristics of participants, vasculitis subtypes, number of patients with osteoporosis, number of patients with fragility fractures, values of bone mineral density (BMD) at the lumbar spine, total hip and femoral neck, and proportion of patients treated with GCs.

The primary outcome was the prevalence of osteoporosis and the prevalence of fragility fractures amongst those with and without vasculitis. The secondary outcomes were the risk for osteoporosis (as reported by the Authors, along with the corresponding classification criteria adopted), the standardized and absolute values of BMD at the lumbar spine, total hip and femoral neck and the risk of fragility fractures of subjects affected by systemic vasculitis as compared to healthy controls, the association between cumulative GC dosage (total grams) and risk of osteoporosis. All the analyses were performed in all the studies, when the data was available.

### Data extraction and quality assessment

Data from studies eligible for the aggregate data meta-analysis were extracted by two authors independently (R.B. and V.M.). Disagreements at this level were resolved by consensus and a third author if needed (E.B.). For all studies, we extracted data on first author, publication year, study design, study country, population characteristics, methods used for osteoporosis diagnosis, type of vasculitis, BMD data, percentage of patients with any fractures and outcomes of interest. In case of multiple publications, we included the most up-to-date or comprehensive information.

Each eligible study was assessed for quality by using the Newcastle-Ottawa scale (NOS) adapted for cross-sectional studies by two independent reviewers (F.P., I.G.), with disagreements resolved through consensus. The NOS uses a star system to evaluate a study in three domains: selection of participants (assigning a maximum of 5 stars), comparability of study groups (assigning a maximum of 2 stars), and ascertainment of outcomes of interest (assigning a maximum of 3 stars). Therefore, 10 stars reflect the highest quality. We judged studies that received a score of ≥8 to be at low risk of bias, studies that scored 7 stars to be at medium risk, and those that scored ≤6 stars to be at high risk of bias.

### Data synthesis and analysis

In order to estimate the prevalence of osteoporosis and fragility fractures amongst those with and without SVs (when available), the number of patients with osteoporosis and fragility fractures amongst all individuals with vasculitis and/or healthy controls was considered as the effect size for each eligible study. Then, these data were pooled and the overall prevalences of osteoporosis and fragility fractures were calculated using a random-effects model. We used the Score (Wilson) method to compute the confidence intervals. To assess the risk of prevalent osteoporosis and fragility fractures in individuals with and without SVs, the odds ratios (ORs) and 95% CIs were also considered as the effect size for each eligible study. In the case of studies reporting several ORs with varying degrees of covariate adjustment, ORs that reflected the maximum extent of adjustment for potential confounding factors, were extracted. The adjusted ORs of all eligible studies were then pooled, and an overall estimate of the effect-size was calculated using a random-effects model, as this methodology considers any differences between studies, even if there is no statistically significant heterogeneity. To assess the difference of BMD in patients with SVs and healthy controls, BMD data were collected from the eligible studies when available and then weighted mean difference was calculated using a random-effects model.

Visual inspection of the forest plot was used to assess statistical heterogeneity. This was also assessed with the I^2^-statistics, which provides an estimate of the percentage of variability across eligible studies that is due to heterogeneity rather than chance alone. Heterogeneity was considered to be low if I^2^ is <25%, moderate if I^2^ is between 25% and 75%, and high if I^2^ is >75% (6). The risk of publication bias was examined using the funnel plot and the Egger’s regression test with logit transformed prevalence of osteoporosis (7).

To explore the possible sources of heterogeneity across the studies and to test the robustness of the observed associations, we performed subgroup analyses by study country, modality of osteoporosis diagnosis, and type of vasculitis.

Univariable meta-regression analyses were also performed to test the effect of specific moderator variables (i.e., age, sex, disease duration and cumulative dose of steroids) on the effect size for the risk of osteoporosis in adults with and without SVs. Finally, we tested for possible excessive influence of individual studies using a meta-analysis influence test that eliminated each of the included studies one at a time.

All statistical tests were two-sided, and p-values of <0.05 (two-tailed) was considered statistically significant. For analyses we used R software (version 4.3.3, R Foundation for Statistical Computing, Vienna, Austria) with “meta” and “metafor” packages.

The protocol of this systematic review was registered in advance on Open Science Framework (DOI: https://doi.org/10.17605/OSF.IO/3G7RJ).

## Results

**Figure 1:**
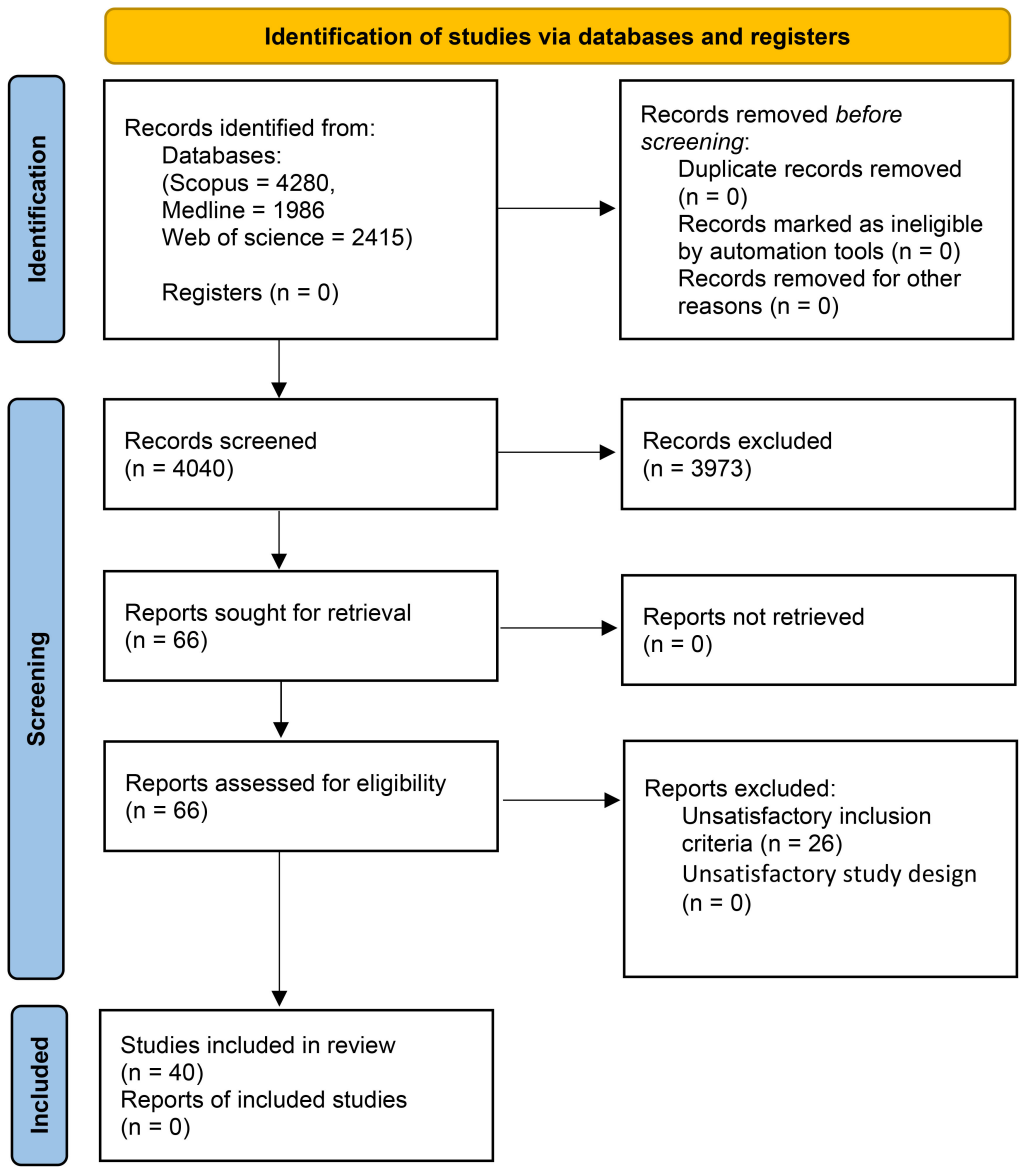
PRISMA flow diagram for the present systematic review.

We identified 66 potentially relevant papers. After examining the full text of these articles, we excluded 26 studies due to unsatisfactory inclusion criteria or unsatisfactory outcome measures (**Supplementary Table 1**). A total of 40 studies were considered eligible for the inclusion in this meta-analysis (**Figure 1**) and were assessed for quality, for a total of 23,358 individuals affected by SVs (mean age 64.8 years, SD: 12.2 years; 39.8% men). **Tables 1** and **2** show the characteristics and RoB of the eligible studies and the characteristics of patients and controls included in the final analysis, respectively. Thiry-three out of 40 studies enrolled patients from hospital-based cohorts and 7 from population-based cohorts.

**Table 1:** Characteristics and RoB of the eligible studies included in the final analysis.

Author (ref)	Publication year	Study characteristics	Population or hospital based	Vasculitis type	Country	Standardized BMD data (T or Z-score)	RoB scale (NOS)
Albrecht K (27)	2018	Longitudinal	Hospital	GCA	Germany	N/A	6
Andersson R (28)	1990	Retrospective	Hospital	GCA	Sweden	N/A	6
Antonini L (29)	2021	Retrospective	Hospital	GCA	France	N/A	6
Bezzerra MC (30)	2005	Cross-sectional	Hospital	Takayasu	Brazil	(BMD only)	7
Bicer A (31)	2004	Cross-sectional	Hospital	Behcet	Turkey	T, Z-score (and BMD)	7
Boomsma MM (32)	2002	Cross-sectional	Hospital	AAV	The Netherlands	Z-score (and BMD)	7
Byung-Woo Y (33)	2021	Cross-sectional	Hospital	AAV	Korea	T, Z-score (and BMD)	7
Cetin B (34)	2021	Cross-sectional	Hospital	AAV	Turkey	(BMD only)	7
De Boysson H (35)	2022	Cross-sectional	Hospital	GCA	France	N/A	5
Eglund M (36)	2016	Cross-sectional	Population	GCA	Sweden	N/A	6
Elgengehy FT (37)	2021	Cross-sectional	Hospital	Behcet	Ecgypt	N/A	5
Faurschou M (38)	2015	Retrospective	Hospital	AAV	Denmark	N/A	5
Harper L (39)	2012	Longitudinal	Hospital	AAV	UK	N/A	8
Henriquez S (40)	2020	Longitudinal	Hospital	Mixed	France	T-score	6
Itabashi M (41)	2012	Longitudinal	Hospital	AAV	Japan	N/A	6
Kermani TA (42)	2018	Longitudinal	Hospital	GCA	US	N/A	6
Lanzillotta M (43)	2020	Retrospective	Hospital	IgG4-RD	Italy	N/A	5
Les I (44)	2015	Retrospective	Hospital	GCA	Spain	N/A	6
Mahr A (45)	2021	Cross-sectional	Hospital	GCA	France	N/A	8
Marco AA (46)	2014	Longitudinal	Hospital	GCA	Spain	N/A	6
Mateo L (47)	1993	Cross-sectional	Hospital	GCA	Spain	N/A	5
Miyano S (48)	2021	Retrospective (nested case-control)	Hospital	AAV	Japan	N/A	8
Mohammad AJ (49)	2017	Retrospective	Population	GCA	Sweden	N/A	5
Myles AB (50)	1975	Longitudinal	Hospital	PMR/GCA	UK	N/A	5
Palmowski A (51)	2022	Cross-sectional	Hospital	Mixed	Germany	N/A	8
Palmowski A (52)	2023	Cross-sectional	Hospital	Mixed	Germany	N/A	7
Paskins Z (53)	2018	Retrospective	Population	GCA	UK	N/A	5
Perrineau S (54)	2021	Retrospective	Hospital	GCA	France	N/A	6
Petri H (55)	2015	Retrospective	Population	GCA	UK	N/A	5
Quartuccio L (56)	2020	Retrospective	Hospital	GCA	Italy	N/A	6
Robson J (57)	2015	Retrospective	Hospital	AAV	Multicentre	N/A	8
Sada K (58)	2020	Longitudinal	Hospital	AAV	Japan	N/A	6
Samson M (59)	2013	Longitudinal	Hospital	Mixed	UK	N/A	6
Sarica SH (60)	2021	Longitudinal	Hospital	AAV	UK	N/A	6
Schmidt WA (61)	2008	Retrospective	Hospital	GCA	Germany	N/A	6
Spiera RF (62)	2001	Prospective	Hospital	GCA	US	(BMD only)	6
Tekin NS (63)	2007	Cross-sectional	Hospital	Behcet	Turkey	(BMD only)	7
Tuckwell K (64)	2017	RCT (cross-sectional analysis of baseline data)	Hospital	GCA	US, Europe	N/A	8
Wilson JC and Sarsour K (65)	2017	Retrospective	Population	GCA	UK	N/A	6
Wilsons JC (66)	2017	Retrospective	Population	GCA	UK	N/A	6

AAV, ANCA-associated vasculitis; BMD, bone mineral density; GCA, giant cell arteritis; IgG4-RD, IgG4-related disease; N/A, not available; RoB, Risk of Bias; NOS, Newcastle-Ottawa Scale; RCT, randomized controlled trial.

**Table 2:** Characteristics of the patients and healthy controls (when available) enrolled in the studies included in the final analysis.

Author (ref)	Vasculitis type	Mean age	N (vasculitis)	% M	% smokers	Disease duration	% GCs treatment	GCs cumulative dosage (g)	% severe kidney involvement	% osteoactive treatment (vasculitis group)	Main findings (vasculitis group): osteoporosis prevalence	Main findings (vasculitis group): fracture prevalence	N (healthy controls)	Control group: osteoporosis prevalence	Control group: fracture prevalence
Albrecht K	GCA	72.7	177	26	13.6	3	78.6			33.1	25.8	7.2			
Andersson R	GCA	78	26	30		5	100	8.2			45		105	38	
Antonini L	GCA	80	101	35	14		100	9.9				24	101		21
Bezzerra MC	Takayasu		30	0		6.96	80						30		
Bicer A	Behcet	38	35	51.4		6.68					6		33	0	
Boomsma MM	AAV	55	99	47.5		4.16	97	10.7			21	8			
Byung-Woo Y	AAV	43.9	35	13			0				8.6		35	0	
Cetin B	AAV	58.5	30	60		5.6	100	11.3		10	23.3	30	20	5	
De Boysson H	GCA		90				100					18			2
Eglund M	GCA	64.5	186	49					19		23	11.8	744	6.58	12.36
Elgengehy FT	Behcet	35	109	89							6.4				
Faurschou M	AAV	59	561	48								11.2	2814		9.9
Harper L	AAV		127			4.3	100					7			
Henriquez S	Mixed	54.4	120	45	8.3	4.56	86	12.6		31	30	27.5			
Itabashi M	AAV	62	30	36		5	100	21.5	100		30	30			
Kermani TA	GCA	71.3	204	24		0.3	92				16				
Lanzillotta M	IgG4-RD	62	131	63		0.41	100	3.5	10		3.8				
Les I	GCA	74	103	34	30	0.5	100					12			
Mahr A	GCA	74	306	35		1.5	100	4.3			6				
Marco AA	GCA	75	106	35		2	100	5.91			52.8				
Mateo L	GCA		56												
Miyano S	AAV	77	149	34	12	0.21	100			55.7		0.6	596		
Mohammad AJ	GCA	76.1	768	25			100				24.5	24	3066	10.2	17.22
Myles AB	PMR/GCA	69	75	25		7	94				9.3	6.6			
Palmowski A	Mixed	67.69	127	66	9.2	5.31	87	13.21		17	19.7	65			
Palmowski A	Mixed	68.1	57	33.3		2.82	100	5.7			10.2	33.3			
Paskins Z	GCA	71.12	2673	28.9	18.7	9.13	83.5			15.45		31.8	10423		10.9
Perrineau S	GCA	74	206	25.7	19	2.8	100			80	11.5	13			
Petri H	GCA		4671				99.67	7			6.25				
Quartuccio L	GCA	70	165	77		4.04	100					11.5			
Robson J	AAV	57.7	535	53.8		1.5	100				14.1				
Sada K	AAV	80	179	38		1	100				8				
Samson M	Mixed	55.6	118	41.5		5	100	4.9	18		16	16			
Sarica SH	AAV	58.7	543	53.6		5.1					5.4		2672	0.8	
Schmidt WA	GCA	69	106	26.5		0.5	100	4.6			28.3	8.5			
Spiera RF	GCA	72.9	21	38		1	100	2.79				19	0		
Tekin NS	Behcet	36.9	30	56		5	0	0	0		3.3		20	3.3	
Tuckwell K	GCA	69	251	25		1	100				18.3	12.4			
Wilson JC and Sarsour K	GCA	72.9	5011	25.9	15.5	5	100				8.86		5011	6.58	
Wilsons JC	GCA		5011			5	100				10.2	8.1			

AAV, ANCA-associated vasculitis; BMD, bone mineral density; GCA, giant cell arteritis; GCs, glucocorticoids; IgG4-RD, IgG4-related disease; M, males; RCT, randomized controlled trial.

The data on the prevalence of GCs was available in 34 studies, with 27 studies including cohorts with a >90% prevalence of steroid treatment. The mean cumulative GC dosage was 7.88 grams, SD 5.22 grams.

### Prevalence and risk of “osteoporosis”.

The criteria for the diagnosis of “osteoporosis” were rather heterogeneous: only 33% of the eligible studies based the definition on dual-energy X-ray absorptiometry (DXA) assessment (of which 13% without reporting the BMD values), with only four studies reporting standardized BMD data. 5% of the studies enrolled subjects based on self-reported history of “osteoporosis”, 50% based on medical records (unspecified criteria), and 12% based on history of fractures extracted from the medical records.

**Figure 2:**
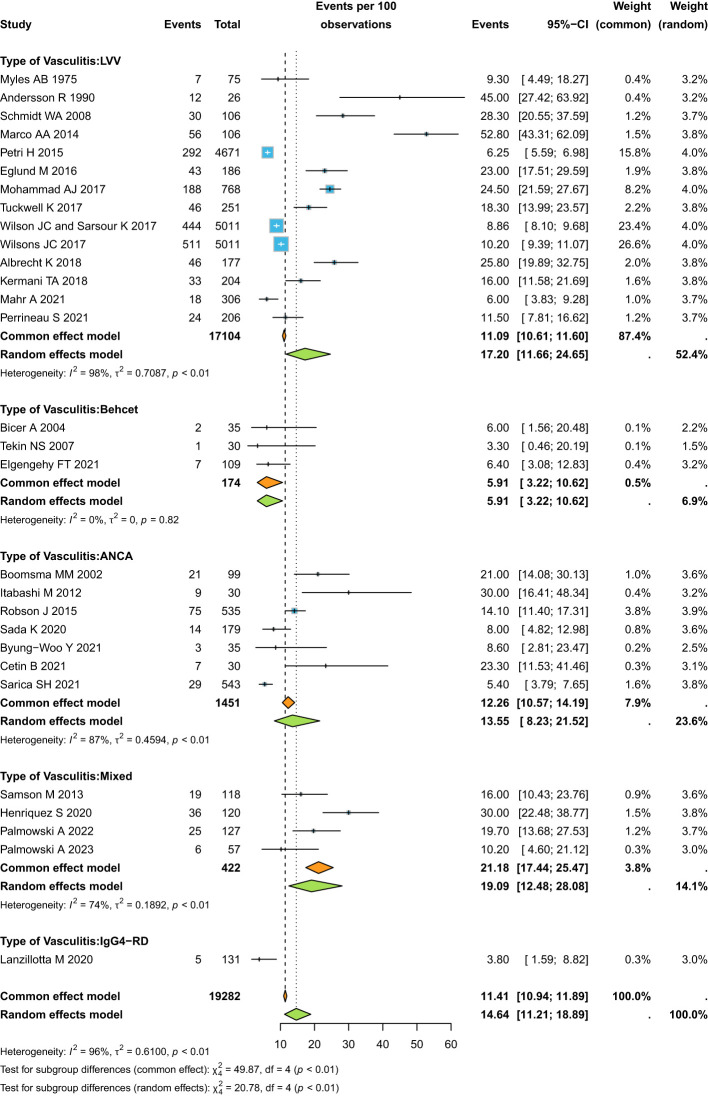
Pooled estimate for the prevalence of “osteoporosis”.

**Figure 3:**
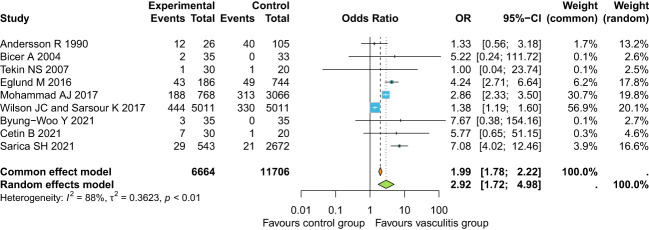
OR of “osteoporosis” in SVs versus healthy controls.

Overall, the pooled estimate for prevalence of “osteoporosis” was 14.64% (95%CI 11.21-18.89) in the whole SVs cohort (**Figure 2**) reporting overall analysis and grouped according to the different types of SVs), with IgG4-RD and Behcet’s being the SVs with the lower estimates and large vessel vasculitides (LVVs), and AAV that with the higher estimate (test for subgroup difference p<0.01). When focusing on the risk of “osteoporosis” in SVs versus healthy controls, the OR was significantly increased: 2.92 (95%CI 1.72-4.98) (**Figure 3**). European and American studies have the higher prevalence of “osteoporosis” as compared to Asia (**Supplementary Figure 1**, results are grouped according to the different countries in which the studies were conducted).

We performed the analysis on the forest plot and pooled estimates for total BMD for the SVs versus control, that yielded non-significant results (weighted mean difference: -0.03, 95%CI -0.07;0.01) (**Supplementary Figure 2**). Given the scarcity of data and unclear technique methodology, it was decided not to proceed with the meta-analysis for Z and T-scores.

Univariable meta-regression analyses to examine the effects of potential moderators on the risk of “osteoporosis”, including age, percentage of men, disease duration and cumulative dose of GCs were performed (**Supplementary Table 2**).

### Prevalence and risk of osteoporotic fractures

**Figure 4:**
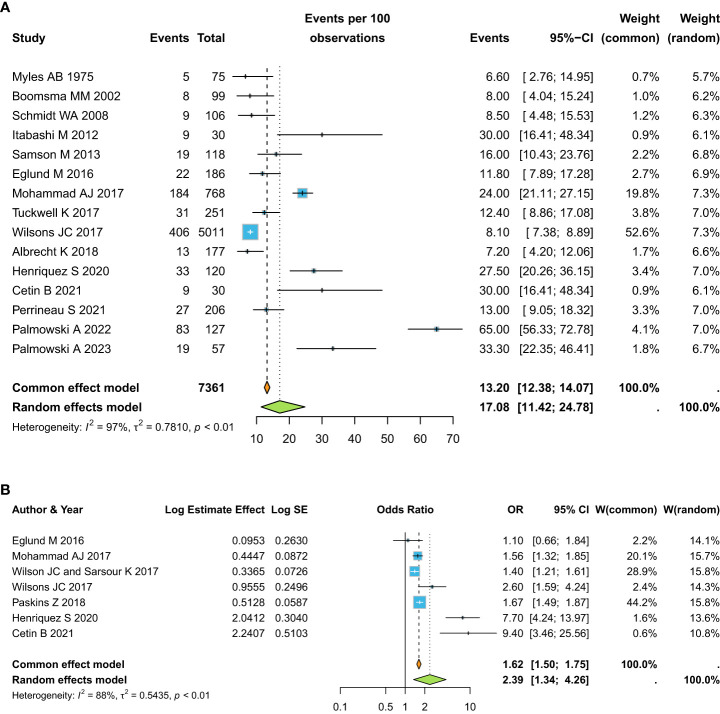
Pooled estimate for the prevalence of osteoporotic fractures in the SVs groups **(a)**, and OR for osteoporotic fractures in SVs versus healthy controls **(b)**.

When focusing on the prevalence of osteoporotic fractures in the SVs groups, we observed an overall prevalence of 17.08% (95%CI 11.42-24.78) (**Figure 4a**). The OR for osteoporotic fractures in SVs versus healthy controls was OR: 2.39 (95%CI 1.34-4.26) (**Figure 4b**).

### Moderator and sensitivity analyses

We found that only the cumulative GCs dosage (total grams) modulated the risk of “osteoporosis” in patients with and without SVs (p=0.0194, estimate = 0.0995, standard error = 0.0426, R^2^ = 0.24), corresponding approximately to an increase of 1% in the prevalence of osteoporosis with each additional gram of cumulative GC. Considering that over the years treatment regimens for SVs have increasingly aimed at reducing the cumulative corticosteroid dose, and given that not all studies reported this data, a sensitivity analysis was performed using an invariable meta-regression analysis with the year of publication as a possible surrogate, and a multivariable analysis including both the year of publication and the cumulative dose of GCs.

The univariable analysis, using the year of publication as a predictor of “osteoporosis” prevalence, was not significant: estimate per publication year -0.015 (95% CI -0.046; 0.154, p=0.32), as was the multivariable analysis: estimate per publication year -0.025 (95% CI -0.083; 0.032, p=0.38) and estimate per GCs cumulative dosage (grams) 0.085 (95% CI -0.004; 0.175, p=0.06).

Regarding fracture prevalence, the univariable analysis was statistically significant: estimate per publication year 0.041 (95% CI 0.022; 0.080, p=0.0022). Similarly, the multivariable analysis also showed statistical significance: estimate per publication year 0.11 (95% CI 0.055; 0.165, p<0.001) and estimate per GCs cumulative dosage (grams) 0.0697 (95% CI -0.0059; 0.145, p=0.070).

In addition, since only 33% of osteoporosis diagnoses appear to be based on DXA and given the potential for misclassification when using patient-reported data or medical records, we performed a sensitivity analysis including only studies that used DXA assessment as an inclusion criterion. The resulting pooled estimate was similar to the original (**Supplementary Figure 3**): 15.91% (95% CI, 9.60–25.51).

### Risk of bias

The risk of bias for each eligible studies was assessed by the NOS tool and is reported in **Table 1**. Overall, there was a moderate-high risk of bias across the studies included in our meta-meta-analysis. In addition, the Egger’s regression test (p-value=0.0541) did not show any statistically significant asymmetry of the funnel plot, suggesting that the publication bias was unlikely (**Supplementary Figure 4**).

## Discussion

We conducted a systematic review and meta-analysis of observational studies to investigate the prevalence of osteoporosis and osteoporotic fractures among patients with different types of SV. We also examined the odds for either condition between patients with SVs versus healthy controls, when this data was available. Our analysis revealed a considerable prevalence of osteoporosis (14.6%) and osteoporotic fractures (17.1%) in patients affected by SVs, with an increased ORs both for osteoporosis (2.92) and fragility fractures (2.39) To our knowledge, this is the first data summarizing comprehensively (by means of a systematic review and meta-analysis) the prevalence of osteoporosis in SVs.

Osteoporosis and fragility fractures are associated with a significant disease burden, including limited mobility, chronic pain, loss of independence, and reduced quality of life (8). Indeed, available evidence show a remarkable increased risk of mortality following osteoporotic vertebral or hip fractures (9, 10), with a 20% one-year mortality rate after a hip fracture (11).

Several autoimmune rheumatic diseases have been associated with systemic bone loss and fragility, including rheumatoid arthritis (RA), spondyloarthritis (SpA), and systemic lupus erythematosus (SLE) (12–15). While there is good evidence suggesting a direct role for conditions such as RA and SpA (12, 13, 16), net of potential confounders such as GC treatment, the direct role of the rheumatic disease in others, such as SLE, remains unclear (17). This may also be the case for SVs, given the magnitude of GC treatments required in these patients and the relevance of associated complications (e.g., chronic kidney disease/end-stage kidney disease, sarcopenia, prevalence of solid-organ transplants, etc.).

The findings of the meta-regression univariate analysis showed that roughly 1% increase in the prevalence of osteoporosis was associated with each additional gram of cumulative GC dosage. Given that the mean cumulative dose in the studies was approximately 8 grams, this finding confirms the significant role of well-known GC-induced toxicity in this context. However, due to the scarcity of studies reporting other relevant factors associated with impaired bone health, the independent impact of SVs remains unclear. Recent vasculitis trials, including GIACTA for GCA (18), ADVOCATE (19) and PEXIVAS (20) for AAV, have focused on reducing glucocorticoid use to minimize treatment-related complications. The tapering glucocorticoid schemes of these trials have been included in the clinical guidelines and are now clinical practice. For instance, the dose of glucocorticoid recommended by the last update of the 2022 EULAR AAV recommendations (21) advise to target 5 mg prednisolone equivalent/day within 4-5 months as compared to 7.5-10 mg of previous guidelines of 2016 (22). By tapering glucocorticoid doses, these trials aim to reduce long-term risks including osteoporosis and other steroid-induced adverse effects. Our findings, obtained with a sensitivity analyses, do not suggest a decline in the reported prevalence of osteoporosis over time. On the contrary there appears to be an increasing trend, even after adjusting for cumulative GC dose. However, this finding should be interpreted with caution, as it may simply reflect a progressively greater awareness and interest in the comorbidities associated with SVs, including osteometabolic complication, thereby influencing reported prevalence rates.

Interestingly, from the present analysis, the mean prevalence of osteoporosis (14.64%) was lower than that of osteoporotic fractures (17.08%), with largely overlapping confidence intervals. This is a limitation and reflects the heterogeneity of the available studies for the systematic review, using different criteria to define osteoporosis and to collected this data, as subjects with history of fragility fractures are classified as osteoporotic regardless of BMD values (23). This led us to conclude that the published data on osteoporosis likely underestimate its true prevalence, which may indeed be higher in patients with SVs.

Additionally, within the general population, approximately 6% of men and 21% of women aged 50–84 years are classified as having osteoporosis according to WHO criteria (24). Therefore, it is unexpected for a high-risk population such as the present one to have a comparable prevalence of this condition, especially after observing a significantly increased OR for fractures in the SV population when compared to healthy controls (OR: 2.39). Similarly, when considering only vertebral fractures, data from the European Vertebral Osteoporosis Study (EVOS) have shown age-standardized population prevalence rates of 12.2% for men and 12.0% for women aged 50 to 79 years (25). Considering that the vast majority of included cohorts received large doses of GCs, with expected fracture events occurring in as many as 30–50% of patients receiving chronic GC therapy (26), led us to speculate that the diagnosis of osteoporosis is probably overlooked, and the fragility fractures largely underestimated. This could be explained by the lack of systematic assessment for bone mineral density impairment in SVs, of the recording of fracture events, and of a systematic search for subclinical vertebral fractures.

This study has strengths and limitations. The primary strength of the current study is that this is currently the first assessment on the state-of the art of the present topic, made with a well-established comprehensive approach (i.e. systematic review) to identify all pertinent studies that fulfil pre-defined inclusion criteria.

Among the limitations, we acknowledge that most eligible studies have relatively small sample sizes. Second, the group of SVs include patients with different forms of vasculitis, resulting in a very heterogeneous group in terms of vasculitis forms and consequently treatment types. To compensate this potential bias, we performed subanalyses based on vessels size, assessing the outcome on more homogeneous groups of SVs. Third, treatments changed significantly over time, including glucocorticoid regimens. Since our study extend on more decades, we performed subanalyses to assess for any time-trends, without significant impact on the outcomes.

Fourth, the methodologies employed for assessing BMD and investigating prevalent fractures are heterogenous and in general not always standardized. Third, the underreporting of significant risk factors such as family history, vitamin D status, kidney function, and other chronic conditions, as well as relevant biochemical data (i.e. bone turnover markers), may have impacted on our findings. Last, data focusing on fracture incidence over time following the diagnosis of SVs are lacking, and the article included in this specific analysis is low.

## Conclusions

In conclusion, the current systematic review and meta-analysis provides a comprehensive overview of the prevalence of osteoporosis and osteoporotic fractures in SVs, indicating that SVs are associated with an increased risk of skeletal fragility. Moreover, this study points out a clear dose-dependence of cumulative GC and risk of osteoporosis.

It remains unclear to what extent this risk is attributed to the SV itself or influenced by subsequent treatments, advanced age, female predominance, and other comorbidities, suggesting the need for further research to investigate these aspects using rigorous methodology. Given the substantial burden associated with fragility fractures, it is imperative to prioritize pre-emptive screening, diagnosis, and treatment of osteoporosis in patients with SVs, including the identification of patients at risk. If all the subjects affected by SVs should or should not undergo to DXA and calcium-phosphate metabolism exam at baseline to avoid porotic fractures needs to be tested in future studies.

## Data Availability

The original contributions presented in the study are included in the article/**Supplementary Material**. Further inquiries can be directed to the corresponding author.
