# Supplementary material for: Osteoporosis and fractures in systemic vasculitides: a systematic review and meta-analysis
Source: Front Immunol. 2025 Mar 17;16:1545546. doi: 10.3389/fimmu.2025.1545546 (PMC11955673; doi:10.3389/fimmu.2025.1545546)
Supplement: Supplementary file 1 [file DataSheet1.pdf]

Search strategy

Pubmed

(((((((((((Churg-Strauss Syndrome[MeSH Terms]) OR (Granulomatosis with Polyangiitis[MeSH Terms])) OR (microscopic polyangiitis[MeSH Terms])) OR (Anti-Neutrophil Cytoplasmic Antibody-Associated Vasculitis[MeSH Terms])) OR (Antibodies, Antineutrophil Cytoplasmic[MeSH Terms])) OR ("EGPA"[tw])) OR ("GPA"[tw])) OR ("wegener"[tw])) OR ("MPA"[tw]) OR “vasculit*”[tw] ((takayasu arteritis[MeSH Terms]) OR ("takayasu"[tw]) OR (vasculitis[MeSH Terms]) OR (giant cell arteritis[MeSH Terms]) OR ("GCA"[tw]) OR ("Horton"[tw]) OR (aortitis[MeSH Terms])) OR ((Polyarteritis Nodosa[MeSH Terms]) OR (“AAV”[tw]) OR ("PAN"[tw]) OR (panarteritis)) OR ((Behcet Syndrome[MeSH Terms]) OR (Behcet’s[tw])) OR ((Immunoglobulin G4-Related Disease[MeSH Terms]) OR (IGG4[tw]) OR (igg-4[tw]) OR (igg4-RD[tw])) OR (((((Cryoglobulinemia[MeSH Terms]) OR (IgA Vasculitis[MeSH Terms])) OR (Vasculitis, Central Nervous System[MeSH Terms])) OR ("LV-GCA"[tw])) OR (Retroperitoneal Fibrosis[MeSH Terms])) OR ("ormond"[tw])))

AND

(((((((((((osteoporosis[MeSH Terms]) OR (bone density[MeSH Terms])) OR (Bone Diseases, Metabolic[MeSH Terms])) OR (absorptiometry, photon[MeSH Terms])) OR (Fractures, Bone[MeSH Terms])) OR ("DXA"[tw])) OR ("DEXA"[tw])) OR ("VFA"[tw])) OR ("frax"[tw])) OR ("giop"[tw])) OR ("BMC"[tw])) OR (skeleton[MeSH Terms])))) NOT case reports[publication type]

Scopus

(TITLE-ABS-KEY (vasculitis) OR TITLE-ABS-KEY (aav) OR TITLE-ABS-KEY (churg-strauss) OR TITLE-ABS-KEY (egpa) OR TITLE-ABS-KEY (gpa) OR TITLE-ABS-KEY (anti-neutrophil AND cytoplasmic AND antibody) OR TITLE-ABS-KEY (anca) OR TITLE-ABS-KEY (granulomatosis AND with AND polyangiitis) OR TITLE-ABS-KEY (eosinophilic AND granulomatosis AND with AND polyangiitis) OR TITLE-ABS-KEY (wegener) OR TITLE-ABS-KEY (microscopic AND polyangiitis) OR TITLE-ABS-KEY (mpa) OR TITLE-ABS-KEY (takayasu) OR TITLE-ABS-KEY (arteritis) OR TITLE-ABS-KEY (aortits) OR TITLE-ABS-KEY (giant AND cell) OR TITLE-ABS-KEY (horton) OR TITLE-ABS-KEY (temporal AND arteritis) OR TITLE-ABS-KEY (gca) OR TITLE-ABS-KEY (polyarteritis) OR TITLE-ABS-KEY (panarteritis) OR TITLE-ABS-KEY (behcet) OR TITLE-ABS-KEY (igg4-related) OR TITLE-ABS-KEY (igg4) OR TITLE-ABS-KEY (igg-4) OR TITLE-ABS-KEY (igg4-rd) OR TITLE-ABS-KEY (cryoglobulinemi*) OR TITLE-ABS-KEY (iga AND vasculitis) OR TITLE-ABS-KEY (schonlein) OR TITLE-ABS-KEY (henoch)) AND (TITLE-ABS-KEY (osteoporosis) OR TITLE-ABS-KEY (osteopenia) OR TITLE-ABS-KEY (bmd) OR TITLE-ABS-KEY (bone AND mineral AND density) OR TITLE-ABS-KEY (bone AND mass) OR TITLE-ABS-KEY (dxa) OR TITLE-ABS-KEY (dexa) OR TITLE-ABS-KEY (fracture)) AND (LIMIT-TO (SUBJAREA , "MEDI")) AND (LIMIT-TO (EXACTKEYWORD , "Human") OR LIMIT-TO (EXACTKEYWORD , "Humans")) AND (LIMIT-TO (DOCTYPE , "ar"))

Web of Science

(TS=("Churg-Strauss Syndrome" OR "Granulomatosis with Polyangiitis" OR "microscopic polyangiitis" OR "Anti-Neutrophil Cytoplasmic Antibody-Associated Vasculitis" OR "Antibodies, Antineutrophil Cytoplasmic" OR "EGPA" OR "GPA" OR "wegener" OR "MPA" OR “vasculit*” OR "takayasu arteritis" OR "takayasu" OR "vasculitis" OR "giant cell arteritis" OR "GCA" OR "Horton" OR "aortitis" OR "Polyarteritis Nodosa" OR “AAV” OR "PAN" OR "panarteritis" OR "Behcet Syndrome" OR "Behcet’s" OR "Immunoglobulin G4-Related Disease" OR "IGG4" OR "igg-

4" OR "igg4-RD" OR "Cryoglobulinemia" OR "IgA Vasculitis" OR "Vasculitis, Central Nervous System" OR "LV-GCA" OR "Retroperitoneal Fibrosis" OR "ormond"))

AND

(TS=("osteoporosis" OR "bone density" OR "Bone Diseases, Metabolic" OR "absorptiometry, photon" OR "Fractures, Bone" OR "DXA" OR "DEXA" OR "VFA" OR "frax" OR "giop" OR "BMC" OR "skeleton"))

NOT

(DT="Case Report")

Supplementary tables and figure legends

Author	Year	PMID	Journal	Reason
Angeli A	2006	16574519	Bone	Population
Bahlas S	1998	9458211	J Rheumatol	Population
Cacoub P	2001	11708421	J Rheumatol	Outcome
Calvo L	2010	20300753	Rheumatol Int	Population
Castan P	2022	35207305	J Clin Med	Outcome
Chanter IW	2003	12634236	Ann Rheum Dis	Population
Cortet B	1999	10339777	Rev Rhum Engl Ed	Population
Daien C	2019	29654949	Joint Bone Spine	Population
Dalbeth N	2002	12088349	Intern Med J	Outcome
Della-Torre E	2021	34144386	Semin Arthritis Rheum	Outcome
Emamifar A	2021	33737697	Sci Rep	Population
Emamifar A	2015	26491449	Int J Rheumatology	Population
Haley JH	1996	8998681	Calcif Tiss Int	Population
Haugeberg G	2000	10898068	Scand J Rheumatol	Population
Huston KA	1978	626444	Ann Int Med	Outcome
Kirnap M	2010	20635679	Bratisl Lek Listy	Outcome
Kupersmith MJ	2001	11756857	J Neuroophthalmol	Outcome
Mo L	2021	34692838	Biomed Res Int	Outcome
Nesher J	1994	7966070	J Rheumatol	Outcome
Ng X	2013	23981747	Int J Rheum Dis	Population
Nordborg E	1997	9408064	J Int Med	Outcome
Rubinow A	1994	6712068	Ann Ophthalmol	Population
Ryan JG	2004	15732231	Ir J Med Sci	Population
Thomas TP	1984	6698408	Gerontology	Population
Unizony S	2015	25667435	Rheumatology	Outcome
Yang S	2021	34239438	Front Pharmacol	Outcome

Supplementary table 1: List and reason for the exclusion of originally screened paper after full-text review.

**Univariate meta-regression analyses**	**Estimates (standard error)**	**P values**
Age (years)	0.024 (0.0139)	0.119
Percentage of men (%)	-0.0138 (0.0098)	0.159
Disease duration (years)	-0.0163 (0.0182)	0.668
Cumulative dose of steroids (total grams)	0.0995 (0.0426)	0.019

Supplementary table 2: Univariate meta-regression analyses to test the effect of age, sex, disease duration and cumulative dose of steroids on the prevalence of osteoporosis in adults with vasculitis.

Supplementary figure 1: PRISMA flowchart of the systematic literature review results and study selection.

Supplementary figure 2: Forest plot and pooled estimates for the prevalence of "osteoporosis" grouped according to the different countries.

Supplementary figure 3: Forest plot and pooled estimates for BMD for the SVs versus control.

Supplementary figure 4: Funnel plot for the evaluation of publication bias.
