# Supplementary figures and images for: Osteoporosis and fractures in systemic vasculitides: a systematic review and meta-analysis
Source: Front Immunol. 2025 Mar 17;16:1545546. doi: 10.3389/fimmu.2025.1545546 (PMC11955673; doi:10.3389/fimmu.2025.1545546)

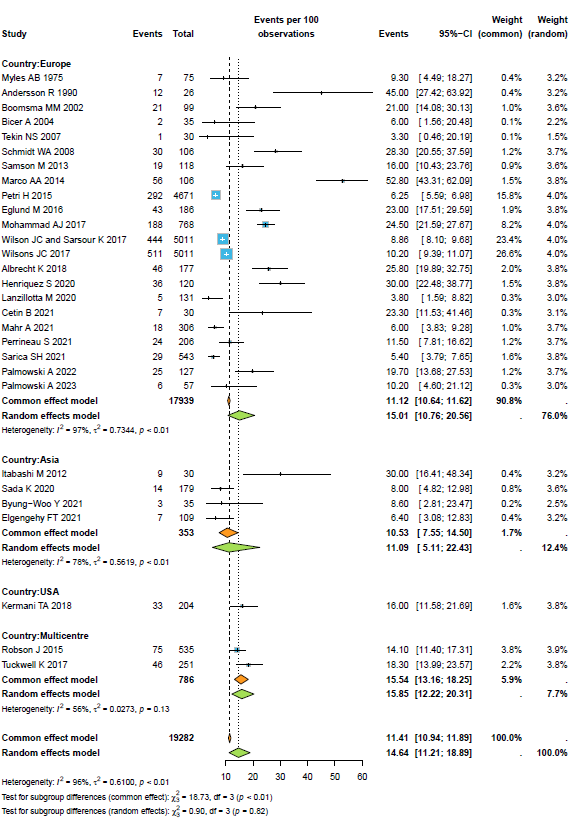

Supplement: Supplementary file 2 [file Image1.tif]

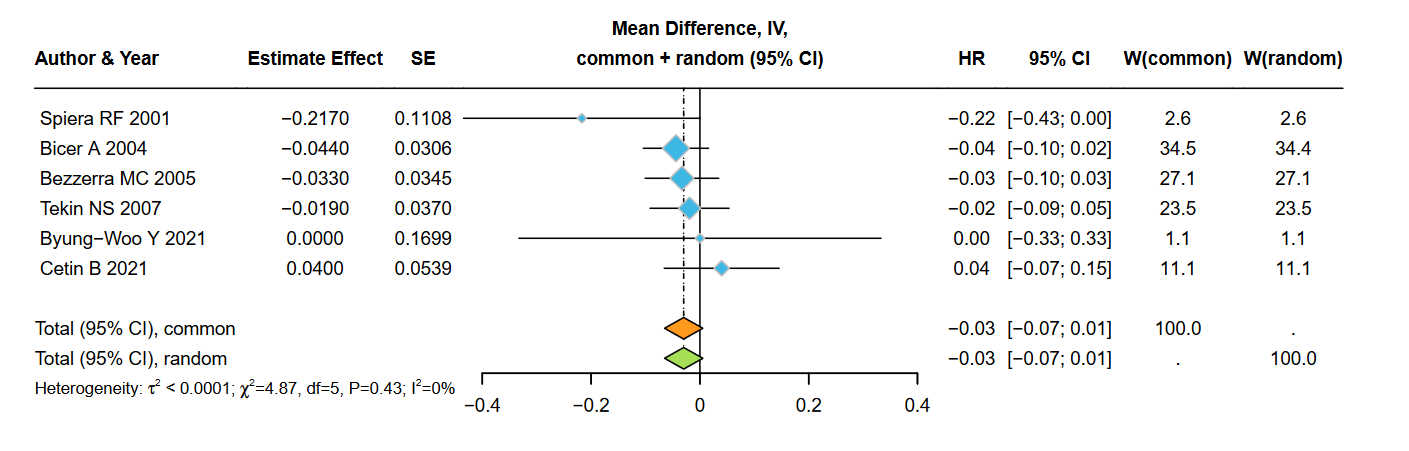

Supplement: Supplementary file 3 [file Image2.tif]

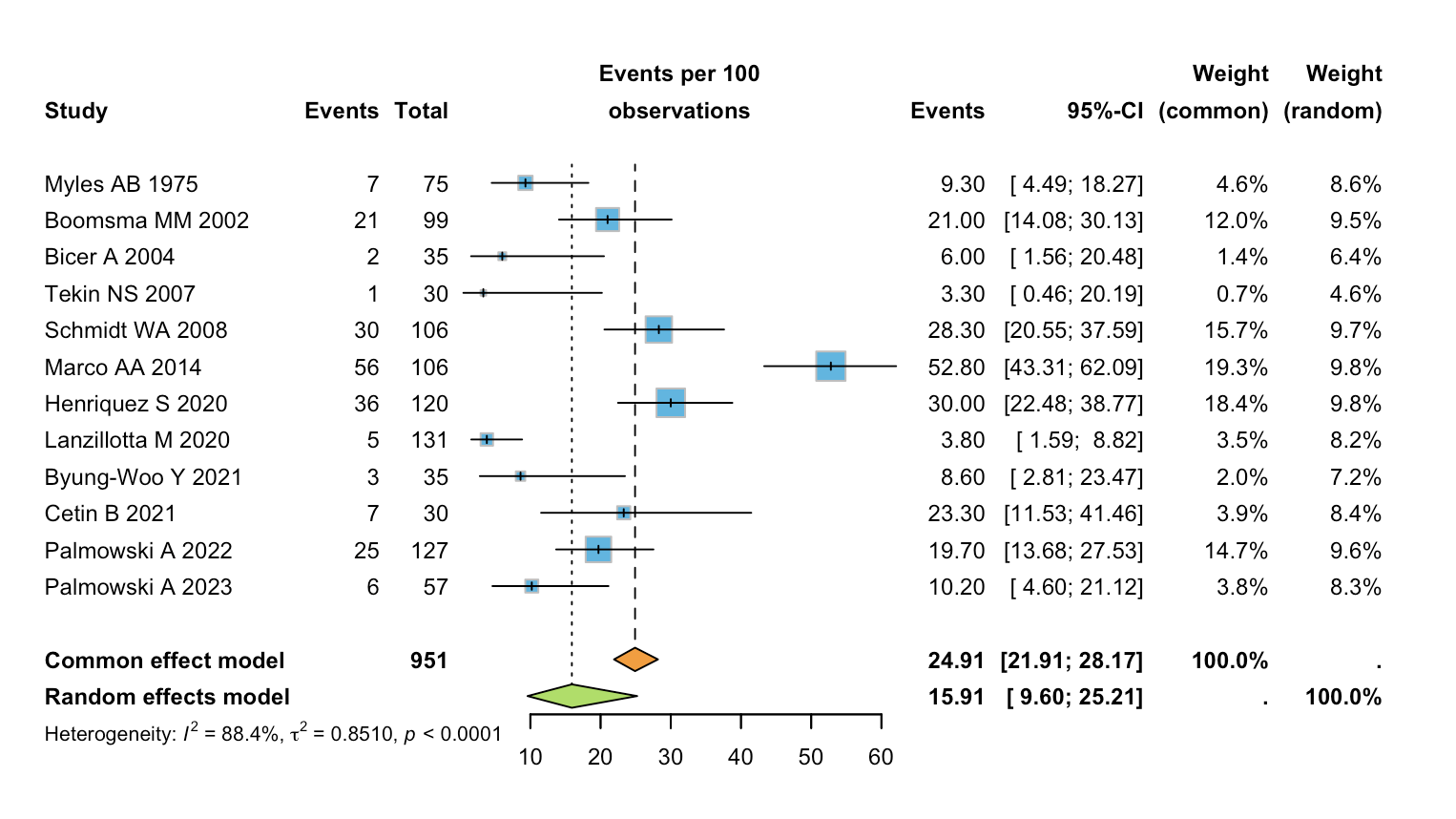

Supplement: Supplementary file 4 [file Image3.tif]

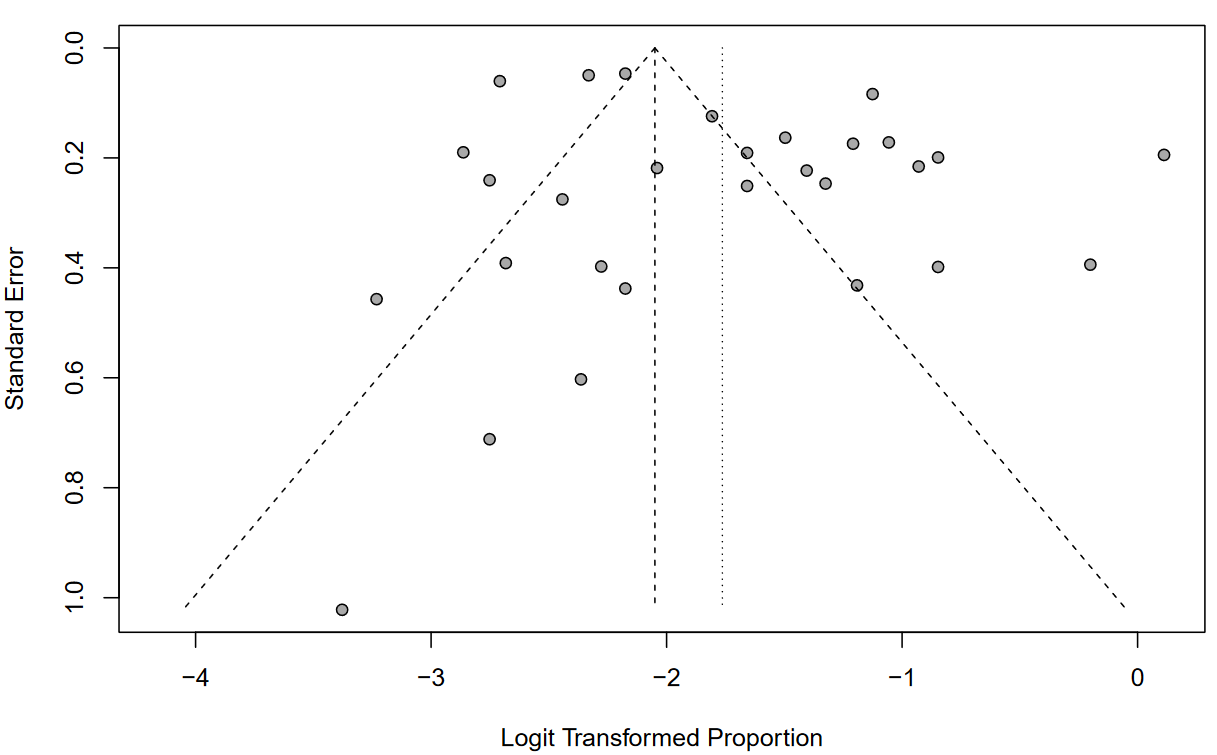

Supplement: Supplementary file 5 [file Image4.tif]
